# ECO-SCORE: Development of a New Ultrasound Score for the Study of Cystic and Solid-Cystic Adnexal Masses Based on Imaging Characteristics

**DOI:** 10.3390/biomedicines13020317

**Published:** 2025-01-29

**Authors:** Carmen Rodríguez-Rubio, Sara Vegas-Viedma, Malena del Olmo-Reillo, Paula Quintana-Zapata, Javier Sancho-Sauco, Mª Jesús Pablos-Antona, Juan Luis Alcázar, Irene Pelayo-Delgado

**Affiliations:** 1 Department of Obstetrics and Gynecology, 12 de Octubre Universitary Hospital, 28041 Madrid, Spain; carmen.rodriguezrubio@salud.madrid.org; 2 Foundation for Biomedical Investigation of Ramón y Cajal Universitary Hospital (FibIO), 28034 Madrid, Spainmalena.del.olmo@gmail.com (M.d.O.-R.); pquintanaz@salud.madrid.org (P.Q.-Z.); 3 Department of Obstetrics and Gynecology, Ramón y Cajal Universitary Hospital, Alcalá de Henares University, 28034 Madrid, Spain; jsanchosauco@gmail.com (J.S.-S.);; 4 Department of Obstetrics and Gynecology, Hospital QuirónSalud, 29004 Málaga, Spain; jlalcazar@unav.es; 5 Faculty of Medicine, University of Navarra, 31008 Pamplona, Spain

**Keywords:** transvaginal ultrasound, adnexal masses, ovarian tumors, score, IOTA, O-RADS, ADNEX model, diagnosis, solid-cystic, cystic masses

## Abstract

The accurate diagnosis of adnexal masses is a critical challenge in gynecological practice. Current ultrasound-based models, such as the ADNEX model, IOTA Simple Rules, and O-RADS, have demonstrated good diagnostic performance but are limited by the inclusion of demographic factors and solid confounding lesions. This study aimed to develop and validate a novel ultrasound score (ECO-SCORE) for cystic and solid-cystic lesions based solely on imaging characteristics to improve diagnostic accuracy and applicability in clinical practice. **Methods:** We conducted a retrospective study on 330 women diagnosed with adnexal masses, including 251 benign and 79 malignant cases. Ultrasound features were analyzed using logistic regression to identify key predictors of malignancy. A new scoring model was developed, excluding demographic or tumor-marker data. Diagnostic performance metrics, including sensitivity, specificity, AUC, and odds ratios, were calculated and compared to existing models using a testing set (20% of the data). **Results:** The ECO-SCORE achieved an AUC of 97.08%, outperforming ADNEX model (87.5%), IOTA Simple Rules (85.7%), and O-RADS (87.5%). Sensitivity and specificity were 92.98% and 88.88%, respectively, with an odds ratio of 106. Key predictors included irregular contour, absence of acoustic shadows, vascularization within solid areas, and vascularization of papillae. **Conclusions:** The ECO-SCORE demonstrated superior diagnostic accuracy compared to established models, highlighting its potential as a reliable tool for assessing adnexal masses using ultrasound features exclusively. Further multicenter validation is needed to confirm its robustness across different clinical settings.

## 1. Introduction

Gynecological ultrasound has become the main tool used to study adnexal masses, as it is non-invasive and widely available. However, it can be difficult to differentiate between benign and malignant lesions because image interpretation is subjective and depends on the experience of the sonographer. This complexity is compounded by the histologic heterogeneity of ovarian tumors.

Misclassification of adnexal masses can lead to significant clinical consequences, such as an increase in periodic check-ups for patients or a delay in oncologic treatments in cases of malignancy. Additionally, unnecessary surgeries on benign lesions not only expose patients to surgical risks but may also negatively affect their reproductive and hormonal function. This reinforces the need for an accurate diagnostic approach based on objective and reproducible tools.

Recent studies have shown that subjective assessment by expert sonographers is the best method to differentiate between benign and malignant masses, with sensitivity ranging from 87.8% to 93.9% and specificity values varying from 69.1% to 89% [1,2,3]. However, professionals with sufficient experience are not always available in all centers.

In order to provide less experienced sonographers with useful tools, several ultrasound scoring models have been developed. In 2000, the International Ovarian Tumor Analysis (IOTA) group published a consensus on the terminology, definitions, and measurements to describe the sonographic features of adnexal masses, establishing a standardized language widely used in clinical practice [4].

**Figure 1:**
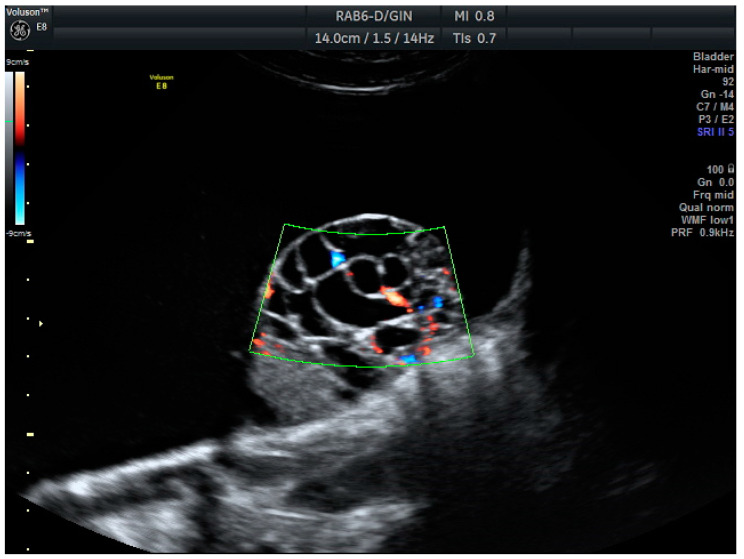
Mucinous carcinoma: Unilocular cystic mass with a solid-cystic papilla inside, showing extensive vascularization (score color 4).

**Figure 2:**
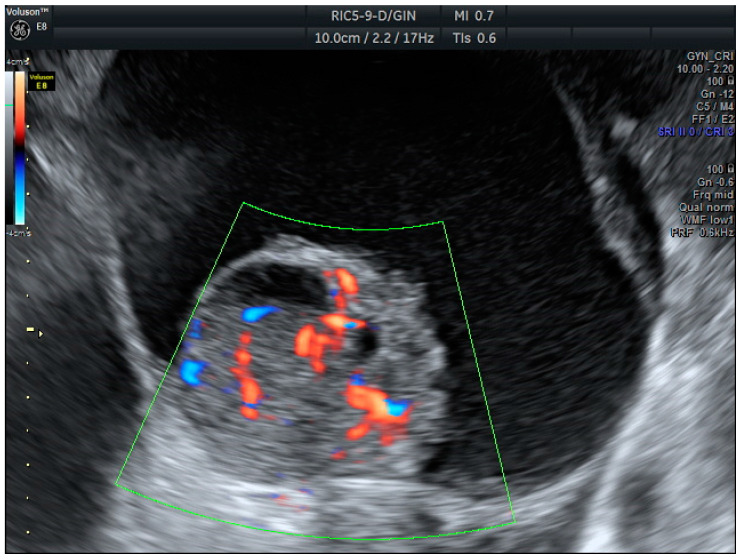
Serous caricinoma. Multicystic mass with a solid papilla inside with a high score Doppler color (score color 4).

Years later, in 2008, the IOTA Simple Rules (SR) were among the first systems to provide a standardized framework for classifying adnexal masses based on ten rules. The benignity features (B) included unilocular cysts (B1) solid components < 7 mm (B2), acoustic shadows (B3), regular multilocular tumor < 100 mm (B4), and negative color maps (B5). The malignancy features (M) contained irregular solid tumors (M1), ascites (M2), ≥4 papillae (M3), multilocular irregular solid tumors > 100 mm (M4), and high vascularization (M5) (Figure 1 and Figure 2). According to SR, masses are classified as malignant if one or more malignancy features are present and no benignity features are observed; benign if one or more benignity features are present and no malignancy features are observed; and inconclusive if both types of features or none are present [5]. These rules have been extensively validated in the literature and have been shown to have good diagnostic performance [6,7]. In 2016, the IOTA Simple Rules Assessment was developed, integrating the Simple Rules with the consideration of whether the patient was treated at an oncology center, improving diagnostic accuracy [8], and adding a percentage of the probability of malignancy.

The ADNEX model (Assessment of Different Neoplasias in the Adnexa) was developed by the IOTA group in 2014, incorporating two clinical predictors (patient age and type of center: oncological/non-oncological), along with six key sonographic values: maximum diameter of the lesion, maximum diameter of the largest solid part, presence of more than 10 locules, number of papillary projections, presence of acoustic shadows, and presence of ascites. Although the use of tumor markers such as CA-125 can be included, evidence suggests that this is not essential in order to have an accurate performance. This model has been validated in multiple studies [9,10,11].

The O-RADS (Ovarian-Adnexal Reporting and Data System) was introduced by the American College of Radiology (ACR) in 2020, based on the lexicon previously defined by the IOTA group for the description of adnexal lesions. O-RADS classifies adnexal masses into six categories (O-RADS 0 as incomplete evaluation and O-RADS 1 to 5 from lower to higher risk), providing not only an estimated risk of malignancy but also specific management guidelines based on each level.

Other diagnostic approaches, such as the Risk of Malignancy Index (RMI) [12] and the Risk of Ovarian Malignancy Algorithm (ROMA) [13], incorporate tumor markers along with clinical and imaging parameters to estimate malignancy risk. The RMI combines serum levels of CA-125, menopausal status, and ultrasound findings into a weighted formula to produce a malignancy score (available at Risk of Malignancy Index (RMI) for Ovarian Cancer). Similarly, ROMA employs CA-125 and HE4 levels, along with menopausal status, in a logistic regression algorithm to classify patients into high or low risk for ovarian cancer (available at ROMA Calculator—XEMA). While these models improve diagnostic specificity in certain populations, they often rely on biochemical markers that can be influenced by benign conditions or demographic variables, potentially limiting their generalizability.

The use of tumor markers such as CA-125 and HE-4 has not demonstrated a substantial improvement in ultrasound performance [14,15]. In fact, multiple studies have highlighted that their systematic application may increase the false-positive rate, which could result in a higher number of unnecessary interventions [16]. These findings support the idea that, in most cases, diagnosis based only on imaging findings is a sufficiently robust tool to differentiate benign from malignant lesions.

Despite the advances made with current ultrasound models, subjective evaluation by an expert sonographer remains the best method to discern between benign and malignant adnexal masses [1,17].

This study proposes the development and validation of a new ultrasound score for cystic or solid-cystic adnexal masses based exclusively on ultrasound features, without the inclusion of tumor markers or other clinical variables. The ECO-SCORE represents a novel approach by focusing exclusively on ultrasound characteristics. This distinguishes it from other widely used models such as ADNEX and O-RADS, which incorporate patient-specific factors.

## 2. Materials and Methods

### 2.1. Study Design

This is a retrospective study of a non-consecutive series of women with a solid-cystic or cystic adnexal mass who underwent surgery at the Department of Gynecology of a tertiary-care university hospital in Madrid (Spain) from January 2021 to June 2024.

The patient’s management was decided according to local clinical protocols and in a multidisciplinary tumor board session. Retrospectively, the clinical information and ultrasound images were reviewed. All patients signed informed consent prior to the inclusion of their data. We obtained approval from the Local Ethics Committee.

### 2.2. Patient’s Inclusion and Exclusion Criteria

The women included had undergone a gynecological ultrasound (no more than 180 days prior) performed by an expert sonographer, with stored images accessible through the hospital’s Picture Archiving and Communication System (PACS, Synapse version 7.4.100, Fujifilm, Mexico) or the ultrasound software. Inclusion criteria included a definitive histological evaluation of the adnexal lesion following WHO criteria [18,19]. Surgical indications were determined based on clinical guidelines, the patient’s medical history, or recommendations from the institution’s oncogynecological committee.

### 2.3. Image Capture

The evaluation ultrasound process began with transabdominal ultrasounds to assess and measure the size of adnexal masses, particularly in cases involving large lesions. This was followed by a transvaginal ultrasound. For patients unable to undergo transvaginal imaging, a transrectal examination was performed. The images were acquired using a RIC 5-9D 4–9 MHz endovaginal probe and a RAB6-D 2–8 MHz transabdominal probe in Voluson E8 (GE Healthcare, Ultrasound, Milwaukee, WI, USA) or Canon Aplio A (Canon Medical Systems Corporation, Tokyo, Japan) ultrasound systems. All images were automatically sent to the hospital’s PACS immediately after acquisition.

Two expert gynecologists with more than 10 years of experience in ultrasound imaging independently reviewed the stored images without knowledge of pathological findings or other imaging results (CT and/or MRI). Disagreements were resolved through further review until consensus was reached. The ultrasound characteristics of adnexal lesions were manually extracted and assessed according to the IOTA Group’s methodology and nomenclature, including Doppler color application [4]. In cases with multiple adnexal masses (bilateral), the most complex lesion was selected for evaluation, or if both were similar, the larger one was chosen.

The features evaluated included the largest tumor diameter (mm), tumor contours (regular or irregular), the presence of acoustic shadows, the presence and size of a solid component, the presence and number of papillary projections (including their dimensions), the presence of septa, the number of locules and ascites, and the Doppler color score (graded 1 to 4) in solid areas, septa, and papillae.

### 2.4. Proposed Scoring

In this study, we proposed a scoring model derived from a formula generated through logistic regression. To achieve this, the process started by incorporating ultrasound (US) features previously identified by Pelayo et al. [2] as indicative of malignancy. These features included the presence of regular or irregular contours, the presence or absence of acoustic shadowing, the size of the solid area, the vascularity of the solid area, the vascularity of the septum, the number of papillae, the size of the largest papilla, and the vascularity of the papillae. Notably, the ECO-SCORE was developed exclusively using ultrasound features without incorporating demographic or clinical variables such as age, menopausal status, or tumor markers. This decision was made to ensure the model’s simplicity and applicability across diverse clinical settings while avoiding potential biases associated with demographic factors.

To assess the impact of each US feature on the likelihood of an adnexal mass being benign or malignant, a logistic regression model was implemented. This method predicts the probability of a binary outcome based on the values of one or more predictor variables. As a result of the logistic regression model, several metrics were extracted. The estimate metric represents the coefficient or weight value calculated for each feature, with a positive value indicating that an increase in the feature value is related to a higher probability of the outcome. The standard error quantifies the variability of the coefficient estimate, with a low value indicating a more precise estimate. The z-value is the test statistic obtained by dividing the estimate by its standard error, allowing for the assessment of significance. The *p*-value indicates the significance of each coefficient, with a value below 0.05 suggesting that the feature significantly contributes to the outcome. The odds ratio reflects how a change in a predictor variable affects the odds of the outcome occurring. Finally, the 95% confidence interval provides a range in which the true odds ratio is likely to fall, spanning from 2.5th percentile to 97.5th.

The entire database was split into 80% for training and 20% for testing. The 20% is reserved for validating the proposed scoring, and the analysis of variables was carried out on the 80% used for training. This proportion is selected following common practices in the machine-learning literature. For most problems, especially for datasets with a low number of samples, 80% of training provides a sufficiently large set to train and fit the model, while the remaining 20% is sufficiently representative to measure performance objectively.

We ensured that the distribution of categorical values was analyzed and balanced in both the training and test sets, in order to avoid overfitting. This procedure minimizes potential biases and enhances the generalizability of the model to unseen data.

### 2.5. Statistical Analysis

RStudio IDE (64-bit version 2023.12.1.402) (Boston, MA, USA) in R programming language was used for data analysis and Excel software for Microsoft 365 MSO (64-bit version 2211) (Redmon, WA, USA) for data recording.

The normality of the distributions was assessed using the Shapiro–Wilk test. This test yields two key metrics: the W statistic and the *p*-value. The W statistic measures how closely the sample distribution aligns with a normal distribution, with a value close to 1 indicating that the data is normally distributed. The *p*-value evaluates the significance of the results; a *p*-value less than 0.05 suggests rejection of the null hypothesis, indicating that the data deviates significantly from normality.

As non-normality was confirmed, the non-parametric Kruskal–Wallis test was selected to assess multicollinearity between numerical and categorical features. This test yields two key metrics: the H statistic (also known as the chi-squared value) and the *p*-value. The H statistic measures the degree of difference among the groups being compared, with higher values indicating greater differences. The *p*-value assesses the statistical significance of the results, with values less than 0.05 typically indicating significant differences between the groups. After performing the Kruskal–Wallis test, eta-squared (η^2^) was calculated to measure the effect size. This measurement shows how strongly the categorical variable affects or relates to the numerical data.

For determining multicollinearity between categorical features, the Fisher’s Exact Test was implemented, which is particularly suited for small sample sizes for expected frequencies less than five in a contingency table. This test provides a *p*-value that indicates if the two categorical features are significantly associated.

For the evaluation of the model with respect to the histological diagnosis, the specificity, sensitivity, accuracy, overall error, precision, F1-Score, positive/negative predictive value, and the corresponding positive/negative likelihood were measured.

## 3. Results

In total, 330 women were included in the present study, with 251 (76%) patients having a benign tumor and 79 (24%) having a malignant tumor. The patients’ mean age and menopausal state were statistically higher in the malignant group (*p* < 0.001). BMI and parity were similar in both groups (Table 1).

In previous studies, Pelayo et al. [2] determined the following US features as significant for malignancy: presence of regular or irregular contour, presence or absence of shadow, size of the solid area, vascularization of the solid area, vascularization of the septum, number of papillae, size of the largest papilla, and vascularization of the papilla.

The complete database was randomly divided into 80% for training purposes and 20% for testing. Hence, 264 patients were selected for the training group, and the remaining 66 patients were used for testing.

To evaluate the influence of each US feature on the likelihood of an adnexal mass being benign or malignant, a logistic regression algorithm was performed. As a result of the analysis, various metrics were obtained per each US feature, including coefficients, standard errors, z-values, *p*-values, odds ratios, and a 95% confidence interval (Table 2). The results highlight that some features significantly influence predicting the probability of malignancy, while others do not show a statistically significant impact. For the “size of the solid area”, “Doppler within septum”, and “Doppler within papillae” features, *p*-values significantly exceed 0.05, suggesting that they may not be significantly influencing the outcome. For the “number of papillae” feature, the *p*-value is equal to 0.05, indicating that a potential effect is happening, but the evidence is not strong enough, so further analysis is needed.

As some features appeared to be non-significant in the outcome probability calculation, a Shapiro–Wilk test was performed to evaluate the normality of the distributions of US features, yielding the W statistic and *p*-value metrics. The “size of the solid area”, “number of papillae”, and “size of papillae” features exhibited W statistics of 0.55, 0.40, and 0.47, respectively, and all had *p*-values of 2.2 × 10^−16^, indicating that they are not normally distributed (Table 3).

The Kruskal–Wallis test was performed to assess the relationship between the numerical and categorical features. The” size of the solid area”, “number of papillae”, and “size of papillae” features exhibited chi-squared values ranging from 255.83 to 260.55, followed by *p*-values below 2.2 × 10^−16^ in all cases, showing a statistically significant relationship between numerical and categorical features. The eta-squared (η^2^) values, which ranged from 0.97 to 0.99, suggest a very strong association between the features, confirming the relevance of these variables for further analysis, as shown in (Table 4).

The Fisher’s Exact test was conducted to evaluate multicollinearity between categorical features. The comparison between “Doppler within solid area” and “Doppler within septum” resulted in a *p*-value of 0.0006124, a value that is significantly below 0.05, indicating a high correlation between both features. Nonetheless, a comparison between “Doppler within papillae” with “Doppler within solid area” and “Doppler within septum” yielded *p*-values of 0.2265 and 0.5076, respectively, suggesting no significant relationship between those features (Table 5).

Based on the results of the statistical analyses conducted, certain features were excluded from further consideration. Regarding Table 2, the” size of the solid area”, “Doppler within septum”, and “Doppler within papillae” features present *p*-values significantly exceeding 0.05, showing no significant influence in the final outcome probability computation. Thus, they were discarded from the final model consideration.

Additionally, certain features were excluded from further consideration based on both statistical analyses and the influence of clinical criteria. For example, the “size of the solid area” and “Doppler within solid area” are highly correlated statistically, as Doppler assessment of the solid area inherently implies their presence (Table 4). Therefore, the “size of the solid area” features were removed, while “Doppler within solid area” features were retained, as they integrated both pieces of information and hold greater clinical significance. The same rationale was applied to features regarding papillae. Given the strong correlation between “size of papillae” and “number of papillae” with “Doppler within papillae”, only “Doppler within papillae” was retained, as it integrated both pieces of information and holds greater clinical significance (Table 4).

Finally, after thorough analysis, the following features were preserved for their statistical significance and clinical impact on the final probability: the presence of regular or irregular contour, the presence or absence of shadow, vascularization of the solid area, and vascularization of the papilla. Subsequently, a new logistic regression model was implemented using the selected features (Table 6). In this subsequent analysis, all *p*-values were below 0.05, indicating that the preserved features had a statistically significant impact on the outcome. Furthermore, all odds ratios were greater than 1, indicating that an increase in the selected features related to a higher probability of the outcome occurring. Moreover, for categorical features with two levels, the odds ratios were higher in the clinically expected level. For instance, a moderate or high Doppler within a solid area is clinically related to a higher risk of cancer compared to a solid area with no or low vascularization.

The diagnostic performance metrics for the proposed scoring are summarized in Table 7, obtained from the validation process over the testing set. The area under the curve (AUC) was 97.08% (91.13–100). The sensitivity was 92.98% (83–98.1), whereas the specificity was 88.88% (51.75–99.72). The positive predictive value (PPV) reached 98.14% while the negative predictive value (NPV) was 66.67%. The odds ratio obtained (OR) was 106.

**Figure 3:**
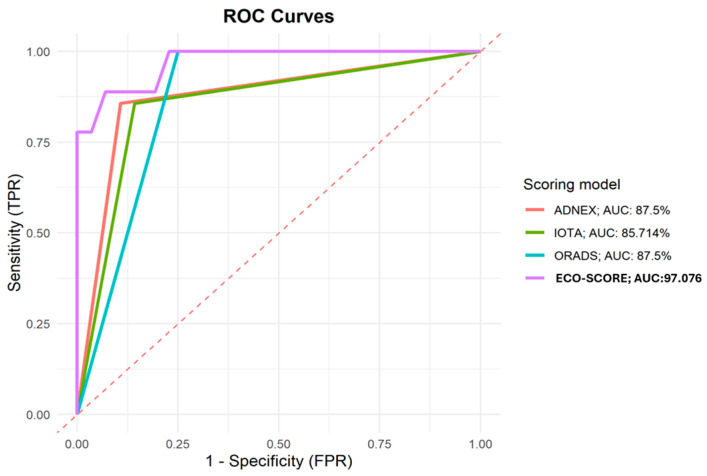
Comparison of area under the curve (AUC) for different approaches (ADNEX model; IOTA Simple Rules Risk Assessment; O-RADS) with the new ECO-SCORE.

**Figure 4:**
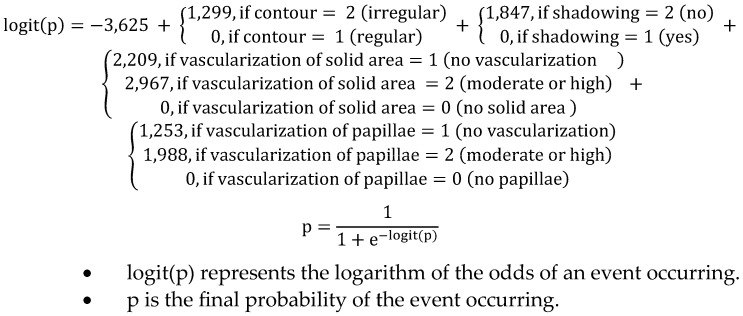
Final formula ECO-SCORE (Registered in Creative Commons Attribution-NonCommercial-NoDerivatives 4.0, number of reference: 2412090328304, available at https://www.safecreative.org/work/2412090328304-eco-score?23, accessed on 11 December 2024).

The ROC curves presented in Figure 3 show the diagnostic performance of our proposed scoring compared to the ADNEX model, IOTA, and O-RADS scoring systems on the testing set. The AUC for our model was 97.08%, while the AUCs for the ADNEX model, IOTA, and O-RADS were 87.5%, 85.7%, and 87.5%, respectively. The ROC curve for our model was visually distinct, positioned above the other models across the range of false-positive rates (FPR), indicating higher sensitivity and specificity. The final formula is represented in Figure 4.

## 4. Discussion

In the present study, a simple scoring model using a logistic regression formula is proposed to differentiate between benign and malignant solid-cystic or cystic adnexal masses. This score was developed exclusively based on ultrasound characteristics previously identified by our group as directly associated with malignancy [2]. Subsequently, we selected the four most statistically relevant features related to malignancy, which included irregular contours, the presence or absence of acoustic shadows, the vascularization of solid areas, and the vascularization of papillae. This approach aims to simplify the diagnosis of adnexal masses while ensuring its applicability for less experienced sonographers.

In cases with bilateral adnexal masses, the largest or most complex lesion was selected for analysis, as this approach maximizes the likelihood of detecting malignancy. While this may occasionally underrepresent smaller lesions, it prioritizes clinical relevance and aligns with current surgical decision-making practices. Moreover, the selection of the most complex or largest mass is a common methodology employed in most similar studies, ensuring consistency with the existing literature and facilitating comparability of results.

To reduce variability in image interpretation and dependence on the expertise of individual sonographers, we implemented standardized imaging protocols based on the recommendations of the IOTA group [4]. These protocols ensure a more uniform assessment of key ultrasound features, thereby minimizing subjectivity in interpretation. Furthermore, emerging tools such as machine-learning algorithms show promise in assisting with feature extraction and image analysis. These technologies can provide diagnostic support, reducing reliance on individual expertise. Although not yet fully validated for widespread clinical use, these advancements represent a step toward standardization and improved diagnostic accuracy.

One of the strengths of the ECO-SCORE is its reliance solely on sonographic features, which eliminates potential biases introduced by clinical or demographic variables such as age or menopausal status. This unique focus enhances its applicability and ensures that diagnostic accuracy is driven exclusively by the imaging characteristics of the lesion.

A higher proportion of benign than malignant masses was obtained in this study, with similar values reported before [2,9]. In relation to the demographic characteristics of our sample, we found associations between older ages and menopausal status with a higher probability of malignancy, as evidenced in the literature [2,3,10,17,20,21]. No differences in malignancy were found in relation to BMI and parity, as has been previously studied [2,22,23,24].

Our model is much simpler, including four sonographic items, than others that have been previously published. For example, the IOTA-SR approach [4] focuses primarily on the type of lesion (categorized as unilocular, multilocular, unilocular-solid, multilocular-solid, or solid), the internal contour of the cyst wall, tumor size, external contour of the tumor, and vascular color score, making a difference between the benign and malignant features, leaving a group of lesions as unclassifiable if they are not included in any of the categories. On the other hand, the ADNEX model incorporates six ultrasound variables (maximum tumor size, maximum size of the solid component, number of locules, number of papillary projections, presence of ascites, and acoustic shadows), two clinical variables (patient’s age and type of center—oncologic or non-oncologic), and one biochemical variable (the absolute value of the CA-125 serum tumor marker) [10] complicating the calculation.

The diagnostic metrics indicate a strong performance of the proposed scoring. The AUC of 97.08% shows an excellent overall diagnostic accuracy, demonstrating the scoring’s ability to discriminate between positive and negative cases. This AUC is notably higher compared to that obtained with the ADNEX model (87.5%), IOTA (85.7%), and O-RADS (87.5%) in our study. These AUCs are consistent with previous studies that validated these scores (88–94% for the ADNEX model [9,16,21,25,26], 87% for IOTA [25], and 86–87% for O-RADS [25,27]).

Using ECO-SCORE, we obtained an overall sensitivity of 92.98 (83–98.1), similar to the previous scores (ADNEX model 90–96, IOTA-SR 93–95, and O-RADS 90.2) [9,10,21,25,26,28]. Nevertheless, we obtained a higher specificity (88.8 versus 64–81.6, 75–86, and 60.5 in the ADNEX model, IOTA-SR, and O-RADS, respectively) [9,10,21,25,26,28]. This demonstrates an improvement in the false-positive rate compared to other models, which would lead to a reduction in unnecessary surgeries, minimizing the associated risks and complications for patients. Additionally, it would decrease patient anxiety caused by false alarms and reduce healthcare costs by avoiding unwarranted interventions and follow-up procedures.

In contrast to other methods that incorporate demographic information such as age and hormonal status, our scoring system focuses exclusively on the sonographic features of the lesions to predict the likelihood of malignancy. Many existing approaches consider factors like age and menopausal status, which can sometimes bias risk assessments, particularly in younger women, by assigning lower risk to premenopausal patients. In 2022, a Cochrane systematic review [29] was conducted with the primary aim of evaluating the accuracy of various diagnostic approaches for ovarian cancer, incorporating menopausal status, ultrasound scans, and biomarkers in both premenopausal and postmenopausal women. The study found that menopausal status could increase the sensitivity of various diagnostic tests for ovarian cancer, including the ADNEX model. However, this same factor was found to reduce specificity, specifically in the premenopausal group. These findings align with other studies [30], highlighting the trade-off between enhanced sensitivity and the increased risk of false positives, which could lead to unnecessary interventions. Relying solely on ultrasound data enables an objective evaluation of adnexal masses, eliminating the potential bias introduced by demographic factors such as age or menopausal status. This represents a significant advantage over other methods, which may inadvertently underestimate the risk of malignancy in younger patients.

On the other hand, the most used biomarker in ovarian cancer is CA-125, which can be elevated in many benign pathologies and physiological situations. It has a sensitivity of 81% and a specificity of 75% to distinguish between benign and malignant masses in postmenopausal patients. However, it has a low sensitivity (50%) in the early stages and a reduced specificity in premenopausal women [29]. HE4, meanwhile, is elevated in 8% of benign conditions compared to 29% for CA-125, improving the specificity, particularly in premenopausal women [29]. Several studies have shown that strategies based solely on imaging diagnosis have good detection rates without the need to use biomarkers [1,15,23,28].

Building on this information and the proven effectiveness of subjective evaluation by expert sonographers [1,17], which is primarily based on ultrasound assessment, we have developed this diagnostic model entirely focused on ultrasound characteristics.

A total of 330 patients were included in the study; however, the use of a non-consecutive sample and the exclusion of certain solid mass types may limit the generalizability of the findings. Solid lesions can often be confusing. A typical fibroma can appear as a non-vascularized solid formation of regular contour with ascites, with a high probability of malignancy if we calculate the risk of malignancy in the ADNEX model, as previously studied [1,2,3]. In contrast, solid malignant lesions will have an irregular contour and appear moderately or intensely vascularized. Therefore, it would be advisable to study the design of another specific score evaluating the characteristics of these lesions. Additionally, patients deemed unsuitable for surgery due to likely benign lesions were excluded, potentially affecting the sensitivity of the diagnostic scores. The ultrasound evaluations were performed at a single center following a standardized protocol for describing adnexal masses, which, while minimizing retrospective study bias, may reduce the applicability of the results to institutions using different protocols. Furthermore, although the model was validated using a separate subset comprising 20% of the dataset, external validation with data from other centers would be valuable to confirm its reliability and applicability across diverse clinical settings. Another limitation of the current study is the lack of explicit testing with artificially added noise to ultrasound images. While the robustness of the ECO-SCORE was demonstrated using real-world clinical images, future studies could explore the impact of simulated noise, such as Gaussian noise, on model performance. This additional validation step could further confirm the reliability of the ECO-SCORE in diverse clinical scenarios and imaging environments.

One potential limitation of this study lies in its retrospective design, which may introduce selection bias due to the inclusion criteria and the reliance on archived ultrasound images. Additionally, the single-center nature of the study may limit the generalizability of the findings, despite efforts to ensure diversity within the dataset. Another potential source of bias could stem from the manual extraction of features, which, although standardized, may be subject to interobserver variability.

Furthermore, our model was developed and validated exclusively on patients who underwent surgery, as histological diagnosis was required as the gold standard. This selection inherently limits its applicability to non-surgical populations, which may not always reflect the surgical cohort in terms of lesion characteristics and malignancy prevalence. Another limitation is that all ultrasound examinations were performed by highly experienced sonographers. This raises concerns about the reproducibility of the ECO-SCORE when applied in clinical settings with less experienced operators. Addressing these limitations, future studies could enhance the generalizability and robustness of our findings.

Additionally, the ECO-SCORE was specifically designed for cystic and solid-cystic adnexal masses, excluding purely solid lesions from its scope of applicability. This limitation reflects the specific focus of the study but restricts the generalization of the model to all types of adnexal masses. Future research should consider extending the methodology to encompass purely solid lesions.

The results related to sensitivity and specificity, while promising, should be interpreted with prudence. The high diagnostic accuracy observed in this study must be corroborated through further research, particularly in prospective randomized controlled trials (RCTs). These trials would offer a higher level of evidence by addressing potential biases and validating the ECO-SCORE in real-time clinical scenarios.

The next step would be to perform a multicenter external validation of the proposed model, using databases from different hospitals and contexts. Such validation could confirm the model’s robustness and applicability across diverse populations, minimizing the risk of bias from any single clinical setting.

To address variability in sonographer expertise, implementing standardized imaging protocols and leveraging emerging technologies such as machine-learning algorithms are promising strategies. These tools can assist in consistent feature extraction and interpretation, potentially reducing the reliance on individual experience.

The shortage of qualified sonographers in some clinical settings poses a significant challenge. This gap could be addressed through strategies such as telemedicine consultations, enabling expert sonographers to interpret images obtained in resource-limited locations. Additionally, combining machine-learning algorithms with standardized imaging protocols may serve as a solution to maintain high diagnostic accuracy even in facilities with fewer resources. These strategies have the potential to expand access to advanced diagnostic tools without compromising the quality of analysis.

## 5. Conclusions

The ECO-SCORE is a simple ultrasound-based score that allows a mathematical and objective differentiation between benign and malignant cystic or solid-cystic adnexal masses, considering only the most important ultrasound features associated with malignancy (irregular contours, absence of acoustic shadows, vascularization of solid areas, and the vascularization of papillae). It has good diagnostic performance compared to other scores and is easier to apply.

However, more studies are needed to show a multicenter validation of the application of the score in different clinical settings.

## Figures and Tables

**Table 1:** Baseline characteristics of the patients in the present study.

Baseline Conditions	Total (N: 330)	Benign (N: 251)	Malignant (N: 79)	*p*-Value
Age (years)	48.4 (SD: 16.39; range 14–91)	46.0 (SD: 16.1; range 14–88)	56.3 (SD: 14.9; range: 14–91)	0.000000514
Menopause				
Yes	142 (43.0%)	88 (35.1%)	54 (68.4%)	0.000000373
No	188 (57.0%)	163 (64.9%)	25 (31.6%)	
Parity				
Nulliparous	137 (42.9%)	102 (41.6%)	35 (47.3%)	0.466
Parous	182 (57.1%)	143 (58.4%)	39 (52.7%)	
BMI (kg/m^2^)	26.0 (SD: 5.17; range 16–45)	26.2 (SD: 4.86; range 16–42)	25.5 (SD: 5.99; range 17–45)	0.421

**Table 2:** Results of the logistic regression model for the US features considered by Pelayo et al. [2].

Coefficients		Estimate	Std. Error	z Value	Pr(>|z|)	Odds_Ratios	2.5%	97.5%
Intercept		−3.84	0.50	−7.67	0.00	0.02	−4.93	−2.95
Irregular contour		1.38	0.54	2.56	0.01	3.96	0.34	2.46
No presence of acoustic shadow		1.62	0.47	3.46	0.00	5.07	0.71	2.56
Size of solid area		0.02	0.01	1.61	0.11	1.02	0.00	0.05
Doppler within solid area	None/Low	1.73	0.75	2.32	0.02	5.65	0.25	3.20
	Moderate/High	1.97	0.84	2.34	0.02	7.15	0.29	3.62
Doppler within septum	None/Low	0.13	0.53	0.24	0.81	1.14	−0.92	1.16
	Moderate/High	1.02	0.83	1.23	0.22	2.78	−0.61	2.65
Number of papillae		0.64	0.33	1.94	0.05	1.90	−0.02	1.29
Size of papillae		0.14	0.06	2.36	0.02	1.14	0.03	0.26
Doppler within papillae	None/Low	−2.17	1.37	−1.59	0.11	0.11	−5.07	0.32
	Moderate/High	−1.27	1.47	−0.86	0.39	0.28	−4.24	1.58

**Table 3:** Results of the Shapiro–Wilk Test for normality of US features.

US Feature	W Statistic	*p*-Value
Size of solid area	0.55	2.2 × 10^−16^
Number of papillae	0.40	2.2 × 10^−16^
Size of papillae	0.47	2.2 × 10^−16^

**Table 4:** Results of the Kruskal–Wallis test for the correlation between numerical and categorical US features.

Numerical Feature	Categorical Feature	H Statistics (Chi-Squared)	*p*-Value	η^2^
Size of solid area	Doppler within solid area	255.83	<2.2 × 10^−16^	0.97
Number of papillae	Doppler within papillae	259.73	<2.2 × 10^−16^	0.99
	Size of papillae	260.55	<2.2 × 10^−16^	0.99
Size of papillae	Doppler within papillae	259.52	<2.2 × 10^−16^	0.99

**Table 5:** Results of the Fisher’s Exact test for correlation between categorical US features.

Categorical Feature	Categorical Feature	*p*-Value
Doppler within solid area	Doppler within septum	0.0006124
Doppler within solid area	Doppler within papillae	0.2265
Doppler within septum	Doppler within papillae	0.5076

**Table 6:** Results of the logistic regression model for the US features selected after analysis.

Coefficients		Estimate	Std. Error	z-Value	*p*-Value	Odds_Ratios	2.5%	97.5%
Intercept		−3.63	0.44	−8.32	0.00	0.03	−4.57	−2.85
Irregular contour		1.30	0.46	2.82	0.00	3.67	0.40	2.22
No presence of acoustic shadow		1.85	0.43	4.30	0.00	6.34	1.02	2.71
Doppler within solid area	None/Low	2.21	0.58	3.83	0.00	9.10	1.08	3.36
	Moderate/High	2.97	0.54	5.53	0.00	19.44	1.95	4.07
Doppler within papillae	None/Low	1.25	0.53	2.36	0.02	3.50	0.21	2.31
	Moderate/High	1.99	0.93	2.13	0.03	7.30	0.20	3.93

**Table 7:** Results of the validation process over the testing set.

Metric	Value
AUC (%)	97.08 (91.13–100)
Sensitivity (%)	92.98 (83–98.1)
Specificity (%)	88.88 (51.75–99.72)
PPV (%)	98.14
NPV (%)	66.67
Odds ratio	106

## Data Availability

The original contributions presented in this study are included in the article. Further inquiries can be directed to the corresponding author.
